# Experimentally recreated workplace environments contain submicron crystalline silica particles, including ultrafine particles, which have been identified in the mediastinal lymph nodes of construction workers

**DOI:** 10.1136/oemed-2025-110330

**Published:** 2026-04-21

**Authors:** Dominique Bazin, Christophe Bressot, Hester Colboc, Laurent Meunier, Hugues Begueret, Adrien Bachmeyer, Ty-Ty Heng-Pradere, Ionut Tranca, Fileto Rodriguez, Frederik Tielens, Ellie Tang, Carine Audouin, Emmanuel Letavernier, Michel Cambrelin, Jean-Francois Bernaudin, Patrick Brochard

**Affiliations:** 1 Université Paris-Saclay, Gif-sur-Yvette, France; 2 Direction Milieux et Impacts sur le Vivant, Institut National de l’Environnement Industriel et des Risques (INERIS), Verneuil, France; 3 INSERM UMRS 1155, Hôpital Tenon Sorbonne Université, Paris, France; 4 Anatomie Pathologique, Hôpital Haut-Lévêque, Pessac, France; 5 Owlo, Paris, France; 6 General Chemistry ALGC – Materials Modelling Group, Vrije Universiteit Brussel, Brussels, Belgium; 7 Service Santé Travail Environnement – Consultation de Pathologie Professionnelle et Environnementale, CHU de Bordeaux Hôpital Pellegrin, Bordeaux, France; 8 Service d’Explorations Fonctionnelle, Hôpital Tenon, Paris, France; 9 Groupement National Multidisciplinaire de Santé au Travail dans le Bâtiment et les Travaux Publics (GNMST BTP), Paris, France; 10 Sorbonne Université Faculté de Médecine, Paris, France; 11 INSERM UMR 1272, Université Sorbonne Paris Nord, Villetaneuse, France; 12 INSERM U1219 – Equipe EPICENE, Université de Bordeaux, Talence, France

**Keywords:** Occupational Health, Chemistry, Inorganic, Construction Industry, Dust, Particulate Matter

## Abstract

**Objectives:**

Although ultrafine particles are suspected to contribute significantly to occupational exposure to crystalline silica, they have not yet been definitively identified in workplaces or human tissues.

**Methods:**

Particles were generated in three 60-minute tests in a glove box: cutting kerb or granite pavement, drilling solid cinder block. The particle concentrations per cm^3^ of air in the box and their mean size were analysed online and using transmission electron microscopy and energy dispersive X-ray analysis (EDX). 17 mediastinal lymph node (MLN) samples from construction workers who underwent lung cancer surgery were examined by Fourier transform infrared spectroscopy (FTIR) and 8/17 by scanning electron microscopy (SEM) with EDX analysis.

**Results:**

The particle concentration was high when cutting kerb or granite pavement (10 000–20 000 cm^−3^ and 50 000–160 000 cm^−3^, respectively), while below 1000 cm^−3^ for drilling cinder block. The aerosol consisted mainly of submicron emissions with a nanometric fraction (ie, <100 nm), showing two main modes: 351 nm to 16 nm for kerb, 407 nm to <16 nm for granite pavement and 310 nm to 17 nm for cinder block processing. Ultrafine silica particles were only identified in kerb and granite cutting samples.

Analysis using FTIR revealed an Si signal in 15/17 of the MLN samples and SEM and EDX analysis detected geometric particle deposition with Si spectra, particularly nano-sized particles, in the eight analysed samples.

**Conclusion:**

Ultrafine silica particles are produced in conditions that mimic typical construction work processes, but the quantity produced varies according to the task. Similar characteristics were observed in ultrafine silica particles in both MLN samples and experimental aerosols.

**WHAT IS ALREADY KNOWN ON THIS TOPIC**

- Using power tools on building materials containing crystalline silica is a major source of respirable crystalline silica emissions.
- The biological response to crystalline silica particles depends on their surface reactivity, particularly that of freshly fractured ultrafine particles of nanometric size.

**WHAT THIS STUDY ADDS**

- An experimental study simulating a mechanical breach of building materials revealed that most of the particles were ultrafine (<100 nm) crystalline silica.
- Ultrafine crystalline silica particles were identified in the mediastinal lymph nodes of construction workers who had been occupationally exposed to silica dust.

**HOW THIS STUDY MIGHT AFFECT RESEARCH, PRACTICE OR POLICY**

- This work highlights the importance of considering the ultrafine fraction of crystalline silica aerosols in dose-response analyses.
- Regulations concerning exposure limit values should consider the size distribution of crystalline silica particles.
- Prevention measures in situations with a high risk of reactive crystalline silica particle emission (eg, freshly fractured particles or ultrafine particles) must be reinforced.

## Introduction

 Crystalline silica is found in a wide range of industrial materials and processes.^1 2^ Inhaling respirable crystalline silica (RCS) particles (ie, particles smaller than 5 µm) at work is a significant health concern,^2 3^ particularly in sectors where the incidence of silicosis is increasing.^4 5^ Workplace exposure to RCS remains a major concern worldwide.^6^ In France, a 2019 study estimated that 365 000 workers were potentially exposed, primarily in the construction industry.^2 7^ Occupational exposure to crystalline silica is regulated through monitoring RCS particles and adopting an occupational exposure limit (OEL) value for RCS dust. In the USA, the permissible exposure limit for crystalline silica has been set at 0.05 mg/m^3^ as an 8-hour time-weighted average across all industries regulated by Occupational Safety and Health Administration since 2016/2017.^8^ In the European Union, the OEL is set at 0.1 mg/m^3^ (8-hour time-weighted average).^9^ In France, the OEL differs for quartz (0.1 mg/m^3^), cristobalite (0.05 mg/m^3^) and tridymite (0.05 mg/m^3^).^10^ A recent review of industrial hygiene literature identified construction tasks involving exposure to RCS.^11^

Some publications have reported the emission of ultrafine (UF, ie, less than 100 nm) particles during tunnel rehabilitation,^12^ road construction or maintenance,^13^ granite polishing,^14^ grinding,^15^ concrete preparation, and treatment^16^ and destruction.^17^ However, these reports do not provide information on whether UF crystalline silica is present. More recent publications have reported the presence of UF crystalline silica particles in experimental studies that reproduce workplace tasks, primarily in relation to processing artificial stone or construction material.^18^,^21^ The role of particle size and surface reactivity in relation to UF silica particles in the pathogenesis of crystalline silica health effects has already been suggested.^18 22^ Although the focus of the information is mainly on the concerns relating to the pathogenesis of manufactured nanoparticles,^23^ including amorphous nano-silica particles,^24^ the common concepts of toxicity can be applied to unintentionally produced UF particles.^25^ Regarding crystalline silica, toxicological data now suggest that nano-sized particles are more reactive than micron-sized particles due to their higher surface-to-mass ratio.^26^,^29^ Moreover, the surface reactivity of UF particles resulting from freshly fractured silica materials is also considered as an important determinant of their toxicity.^22 23^ Therefore, characterising the UF fraction of occupational silica aerosols is potentially an important consideration when assessing health risks to workers.

The primary objective of this study, therefore, was to characterise the emissions of UF crystalline silica particles from stressed materials used in construction activities. To simulate the exposure of workers at the construction site, a small-scale mechanical stress study was conducted in an emissions chamber so that it closely resembled such emissions. The analysis of the silica particles covered the widest possible size range. Second, the results were compared with those observed in the mediastinal lymph nodes (MLNs) of construction workers who have undergone lung cancer surgery and have been occupationally exposed to RCS.^30^

## Materials and method

### Experimental emissions from stressed construction materials

The experimental procedure was designed in collaboration with the French Occupational Health in Construction Industry (GNMST-BTP; https://gnmstbtp.org/). Three types of building material commonly used in construction and known to contain crystalline silica were tested for experimental emissions from stressed construction materials: a concrete kerb, a natural granite pavement slab and a solid cinder block (online supplemental figure 1) (references are listed in table 1).

**Table 1:** Summary of particle emissions from the different samples experimentally tested by cutting (Dremel Disk machine) or drilling (Skil impact drill)

Samples	Test*	Particles emitted during the test					
		Total particle distribution mode (see figure 1B)	Free particles of crystalline silica			Silica containing composite particles	Crystalline silica detection (X-ray analysis)
			[Table-fn T1_FN2]UF silica†	<1 µm	>1* *µm		
Concrete mortar Kerb stone type T2 https://www.Bonomi-beton.fr France 9.5×12×25 cm	Cutting Dremel Disk machine 400 digital, 175 W, 28 000 rpm, cutting disc alumina No. 409 https://www.dremel.com/fr/fr France	Submicronic: (436–351 nm) Nanometric *<*16 nm	+	+	+	+	+
Natural granite pavement https://www.noblema.com/ France 18×11×24 cm		Submicronic (673*–*352 nm) Nanometric <16*–*17 nm	+	+	+	+	+
Solid cinder block https://www.pointp.fr/c/ France 1894065 19×10×49.5** cm**	Drilling Skil impact drill 6280 550 W 0–3000 rpm concrete drill Ø 6 mm. https://www.skileurope.com/fr/ France	Submicronic: (1556–310 nm) Nanometric <16*–*17 nm	–	–	+	+	+

* The test protocols have been developed in collaboration with the GNMST-BTP (https://gnmstbtp.org/) to provide the most realistic description of the work.

† UF-SiO_2_: Ultrafine (ie, <100 nm) particles of crystalline silica.

**Figure 1:**
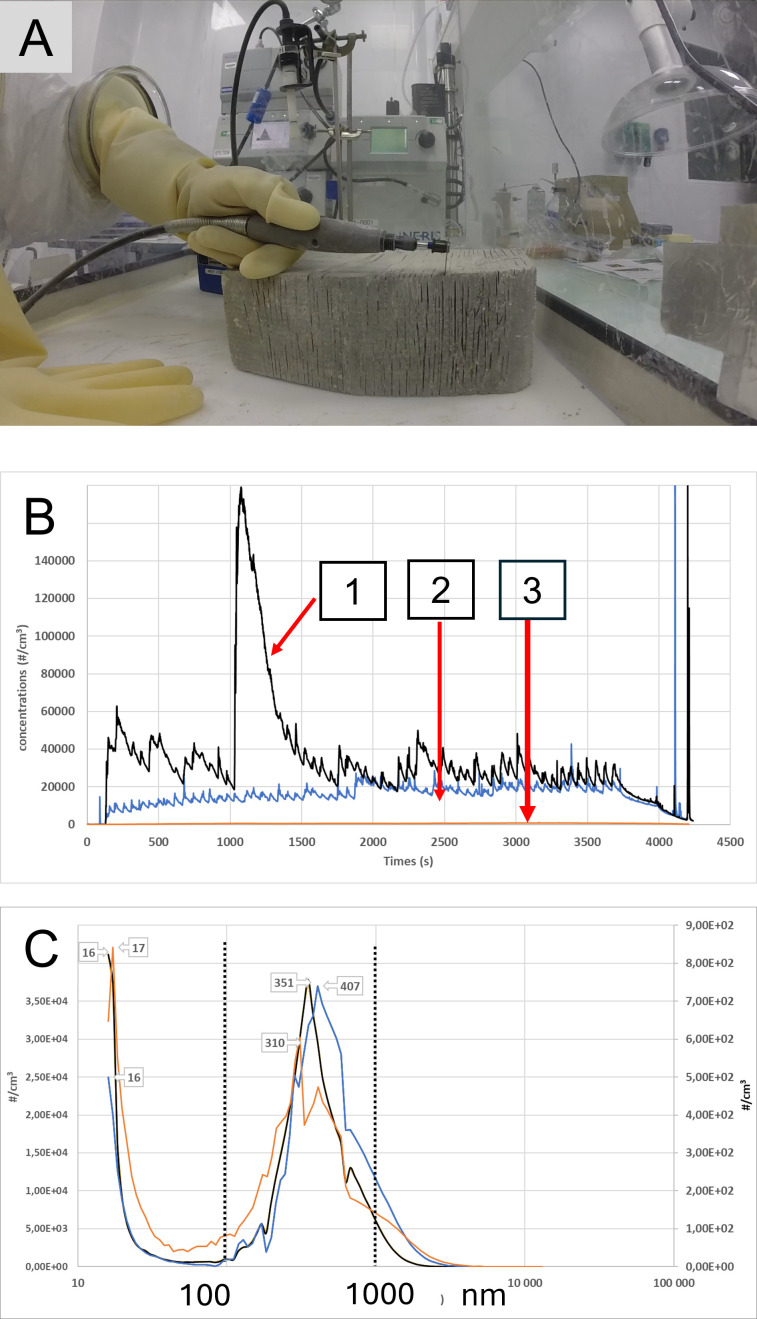
Experimental emissions from stressed construction materials. (**A**) The experimental set-up, that is, a glove box containing a granite pavement slab being cut. The Dremel motor is placed outside the box to prevent emissions during cutting. The three materials that were tested are shown in online supplemental figure 1. (**B**) The average particle emissions using 3775 condensation particle counter from TSI (range 4 nm–2.5 µm) over three 60-minute tests of a concrete kerb (black curve arrow 1); a granite pavement slab (blue curve, arrow 2) cut with a Dremel, and a solid cinder block drilled with a Skil percussion drill (orange curve, arrow 3). The X-axis shows the experimental times and the Y-axis shows the particle concentration (× cm^−3^) which started at 10 000 cm^−3^. (**C**) The size distribution between 15 nm and 20 µm obtained using Aerodynamic Particle Sizer and Scanning Mobility Particle Sizer Spectrometer merge software for the concrete kerb cut (black curve and left scale), the granite pavement (blue curve and left scale) and the solid cinder block (orange curve and right scale) over 60 min. The numbers with arrows on the graph indicate the corresponding particle sizes to the emission peaks. According to the graphs, nanosized particles were present in significant quantities throughout the tests.

These samples were chosen based on GNMST-BTP recommendations and corresponded to materials that are commonly used on construction sites in France. Workplace stress for the first two materials is typically obtained by using disc machines on demolition sites, such as the Dremel disc machine (see table 1 for reference), while the third material is often drilled using a Skil percussion drill (see table 1 for reference) representative of the conditions that would be encountered in a workplace, with this type of material.

The materials were placed in a 317-litre glove box with an air change rate of 1.57 per hour. For cutting operations, the Dremel disc machine was positioned outside the glove box (ie, the emission chamber) using a transfer arm to prevent contamination from the electric motor (figure 1A). Three different tests were completed. The concrete kerb and granite pavement samples were cut with the Dremel to create a groove using a No. 409 (aluminium oxide) cutting disc (see table 1 for reference). Each operation consisted of 10 s of cutting followed by 50 s of waiting. This cycle was repeated for a total duration of 15 min. This sequence of operations was first performed for 30 min, followed by a further 60 min.

In order to drill the solid cinder block, the operator used a Skil percussion drill passing the drill bit through the glove box wall. Each operation consisted of 10 s of drilling followed by 50 s of waiting. This procedure was repeated for a total of 15 min, followed by two further drilling sessions, lasting 30 and 60 min, respectively.

An online particle count of the experimental emissions was performed using a Scanning Mobility Particle Sizer Spectrometer (SMPS) and the TSI Aerodynamic Particle Sizer (APS).^31^ The SMPS combines the TSI 3775 condensation particle counter (CPC) (TSI Incorporated, Minnesota 55126 USA) and the Differential Mobility Analyser (DMA) 3080 (Waters New Castle, DE19720, USA). The TSI 3775 CPC provides a real-time total of particle number concentration, for particles ranging from 4 nm to 3 µm, within a concentration range of 0–10 000 000 #/cm^3^. The DMA 3080 can size particles between 15–750 nm. The APS is designed to size larger particles between 0.5–20 μm. A dilution of 100 was used to protect the APS from high particle concentrations. The size distributions from SMPS and APS presented in this study were systematically averaged over the duration of the three tests using TSI’s Data-Merge software.

**Figure 2:**
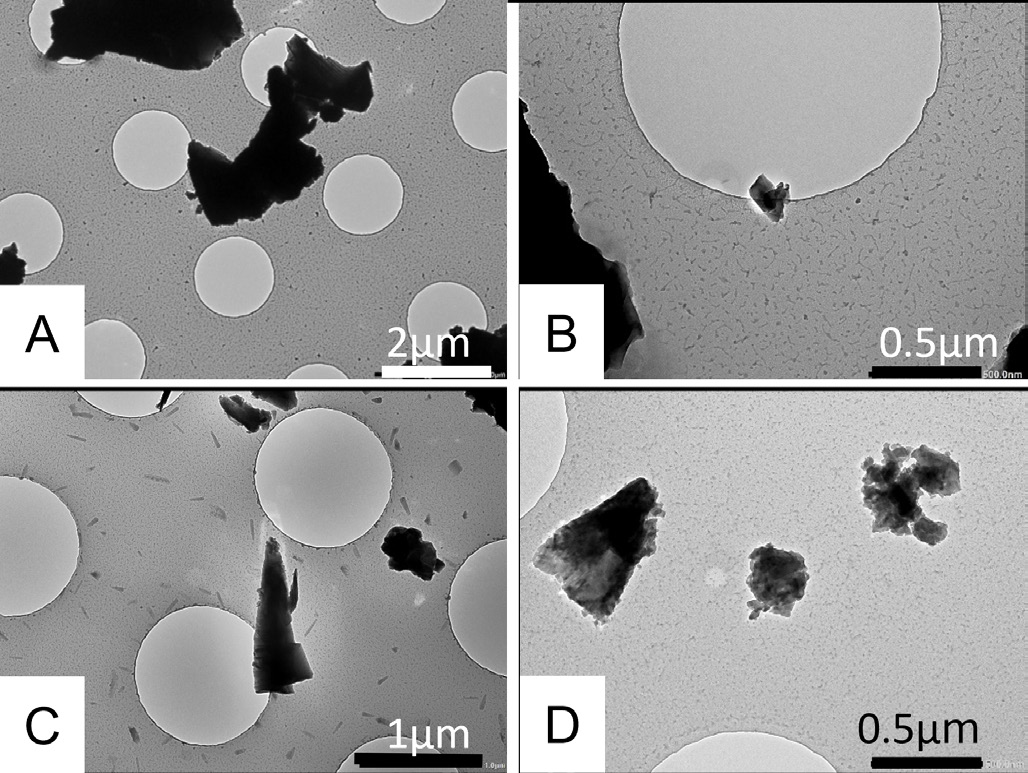
Transmission electron microscopy analysis of particles emitted when construction materials are stressed. (**A**) An overview of the aerosol collected from the concrete kerb under stress. Objects are identified as supermicron (over 1 µm), submicron (under 1 µm) or nanosized (100 nm and under). (**B**) Nano-SiO2 particles collected from concrete kerb stress (Si 48% O 46% w/w). Diffraction analysis of the particle reveals a crystalline structure. (**C**) Particles collected during the cutting of a granite pavement slab with a Dremel tool (Si 50% and O 45% w/w). Diffraction analysis shows crystalline structures. (**D**) Submicron objects collected during cinder block drilling. (Ca 30% and O 29%, Si 23%, K 7%, Al 6% w/w).

To characterise the particles, glove box air samples were collected for a period of 10 s (for the Dremel procedure) and 10 min (for the drilling procedure) at the centre of the load on a porous MET grid using the INERIS Mini Particle Sampler (Ecomesure SAS, Saclay France)^32^ which collects particles without the need to select the aerosol size. Transmission electron microscopy (TEM) imaging was performed on a JEOL JEM-1400 Plus (JEOL Europe Rueil-Malmaison France) operating at 120 kV and equipped with an energy-dispersive spectroscopy microanalysis system (AZTEC Oxford Instruments, les Ulis France) to characterise the size, shape and elemental chemical composition of each particle emission. Sampling was performed in close proximity to the source, to ensure identical real-time measurement conditions. This configuration enabled the physicochemical characterisation of the aerosol by TEM as shown in figure 2.

To identify RCS, samples were also collected on a 25 mm filter coupled to an alveolar particle selector, a DORR-OLIVER cyclone (LAPREV Voisins-le-Bretonneux, France), with an air flow set at 1.7 L/min, in accordance with the Metropol M-158 protocol.^33^ This protocol, which was developed for sampling particles below 10 µm includes analysing the collected particles by gravimetry and X-ray analysis.^33^

### Chemical and morphological characterisation of submicronic and UF crystalline silica particles in mediastinal lymph nodes

This study forms part of a larger investigation of occupational silica exposure among a cohort of French consecutive patients with non-small cell lung cancer, all of whom underwent surgery for lung cancer with systematic MLNs dissection. It comprises a detailed industrial hygiene expertise of each job history, including silica exposure.^30^ The present study was based on the retrospective reporting of occupational and pathological data collected as part of clinical management of these patients. The study focused on submicronic (below 1 µm) inorganic particles (with a UF fraction below 100 nm) in MLNs. 17 paraffin-embedded MLN tissue blocks, which had been annotated as corresponding to patients with occupational exposure to silica in the construction industry, and/or the presence of silica nodules in lymph nodes (which were detected in 13 of the 17 samples) were selected.^30^ 5 µm tissue sections were placed on Low-e microscope slides, which are optical quality glass with infrared reflective coating. They allow visible observations and infrared reflectance measurements (MirrIR, Kevley Technologies, Tinta Sciences, Parma, Ohio, USA). The paraffin was removed using xylene, after which two examination methods were employed: Fourier-transformed infrared (FTIR) spectroscopy was used for the large-scale analysis of tissue sections, while scanning electron microscopy (SEM) coupled to an energy-dispersive X-ray (EDX) spectrometer was used to characterise particles.

FTIR is a well-known non-destructive and label-free technique used to analyse the chemical composition of organic and inorganic materials.^34 35^ An IN10MX microscope (Thermo Fisher Scientific, Waltham, Massachusetts, USA) was used to collect InfraRed (IR) spectra in ultrafast mode with a 50 µm × 50 µm aperture. Spectra were collected in the 4000–800 cm^−1^ mid-infrared range with a resolution of 16 cm^−1^.^34 35^ FTIR analysis was completed on the 17 selected samples.

A subset of eight of these samples was selected for SEM-EDX analysis. This selection was based on documented high silica exposure as determined by the occupational questionnaire or the presence of silicotic nodules,^30^ and concurrent positive FTIR results. Morphological observation of the mineral deposits on the MLN surface was combined with an EDX spectrometry to analyse the elemental composition of the samples by measuring electron-induced X-ray fluorescence.

This analysis was performed using a Zeiss SUPRA55-VP SEM equipped with an Oxford Instruments X-ray (EDX) spectrometer (Carl Zeiss AG, Feldbach Switzerland). This field emission gun microscope operated at an electric potential of 0.5–30 kV. High-resolution observations were made using an Everhart-Thornley detector. Measurements were taken at low voltages, between 0.5–2 kV. Additionally, for each of the eight samples, particles were randomly selected from the SEM data. 10 of these were then chosen for EDX analysis.^36^

## Results

### Silica emissions from stressed construction materials

Figure 1B illustrates the particle counts during the tests, summarising the average particle emissions from three 60-minute tests performed on the concrete kerb (black curve), granite pavement slab (blue curve) and solid cinder block (orange curve). Cutting the concrete kerb with the Dremel disc machine resulted in a particularly high particle concentration, typically between 50 000–160 000 #/particles/cm³. Cutting granite pavement slab with the Dremel disc machine resulted in a particle concentration of between 10 000–20 000 #/particles/cm³. In contrast, the number of particles emitted when drilling solid cinder blocks was much lower than for the previous two materials, at a value below 1000 #/particles/cm³. The black curve (cutting of the concrete kerb) shows short, periodic increases in the number of particles emitted, in relation to the applied stresses. The basic sequence consisted of a short period of cutting followed by a pause. These results must be compared with the initial background noise level of 50–100 particles per cm^3^.

The analysis of the aerosols produced during representative loading periods was carried out throughout 60-minute tests. In the test that consisted of cutting a concrete kerb, the emitted polydisperse aerosol consisted predominantly of submicron emissions (less than 1 µm) (figure 1C, black line). However, a nanometric fraction (100–200 nm and below) was discernible, as well as a small supermicron (over 1 µm) fraction. The size distribution ranged from 16 nm to 4.2 µm, showing two main modes at 351 nm and below 16 nm. The average aerosol produced by cutting the granite pavement slab was also polydisperse, ranging from submicron to weakly supermicron (with a maximum detected size of 8 µm) and contained a substantial nanosized fraction (figure 1C, blue line). Two main modes were discernible at 407 nm and below 16 nm. After drilling a solid cinder block for 60 min, the resulting aerosol exhibited a polydispersity between 15 nm and 4.4 µm, with a significant nanometric component (figure 1C, orange line). The primary and secondary modes of the aerosol were positioned at 17 nm and 310 nm, respectively.

In conclusion, the emission of submicronic particles with peaks at 351 nm, 407 nm and 310 nm was observed for all three materials tested for cutting kerb and pavement slabs and drilling solid cinder, respectively. These results are summarised in table 1. Each X-ray diffraction test provided an integrated sample for determining the crystalline nature and concentration of RCS in glove box air. The average concentrations were based on three separate trials and expressed per cm^3^ of air. Samples obtained during the drilling of solid cinder blocks were the most easily exploitable, yielding quartz and cristobalite concentration values of 0.016 mg/m³ and 0.014 mg/m³, respectively. Samples obtained through cutting sections of granite pavement fell within the calibration curve of the X-ray diffractometer. Concentrations of 7.3 mg/m³ for quartz and 0.039 mg/m³ for cristobalite were obtained. Concentrations systematically exceeded the calibration curve when cutting concrete kerb. Concentrations of 5.3 mg/m³ for quartz and 0.034 mg/m³ for cristobalite were obtained.

TEM examination of the particles emitted, when cutting the concrete kerb, showed that there was a considerable number of particles in the samples and that these varied considerably in size (figure 2). Most of these particles were smaller than 5 µm. A small number of supermicron particles were also found alongside a larger number of submicron or nanosized particles.

Analysis of the particles emitted when granite pavement slab was cut revealed a size distribution of particles ranging from a few nanometres to a few micrometres. Notably, there was a high prevalence of submicron objects. Electron diffraction results indicated that these particles were crystalline in nature. The sample grids revealed an abundance of crystalline silica objects, including nanometric silica crystals.

When drilling a solid cinder block, the collected particles were predominantly submicron and consisted of high concentrations of calcium. Electron diffraction experiments indicated the presence of crystalline structures within the matrix. Despite the prevalence of nanoparticles in the samples, no free silica nanoparticles were observed. The results are summarised in table 1.

### Characterisation of submicronic and UF crystalline silica particles in MLN

**Figure 3:**
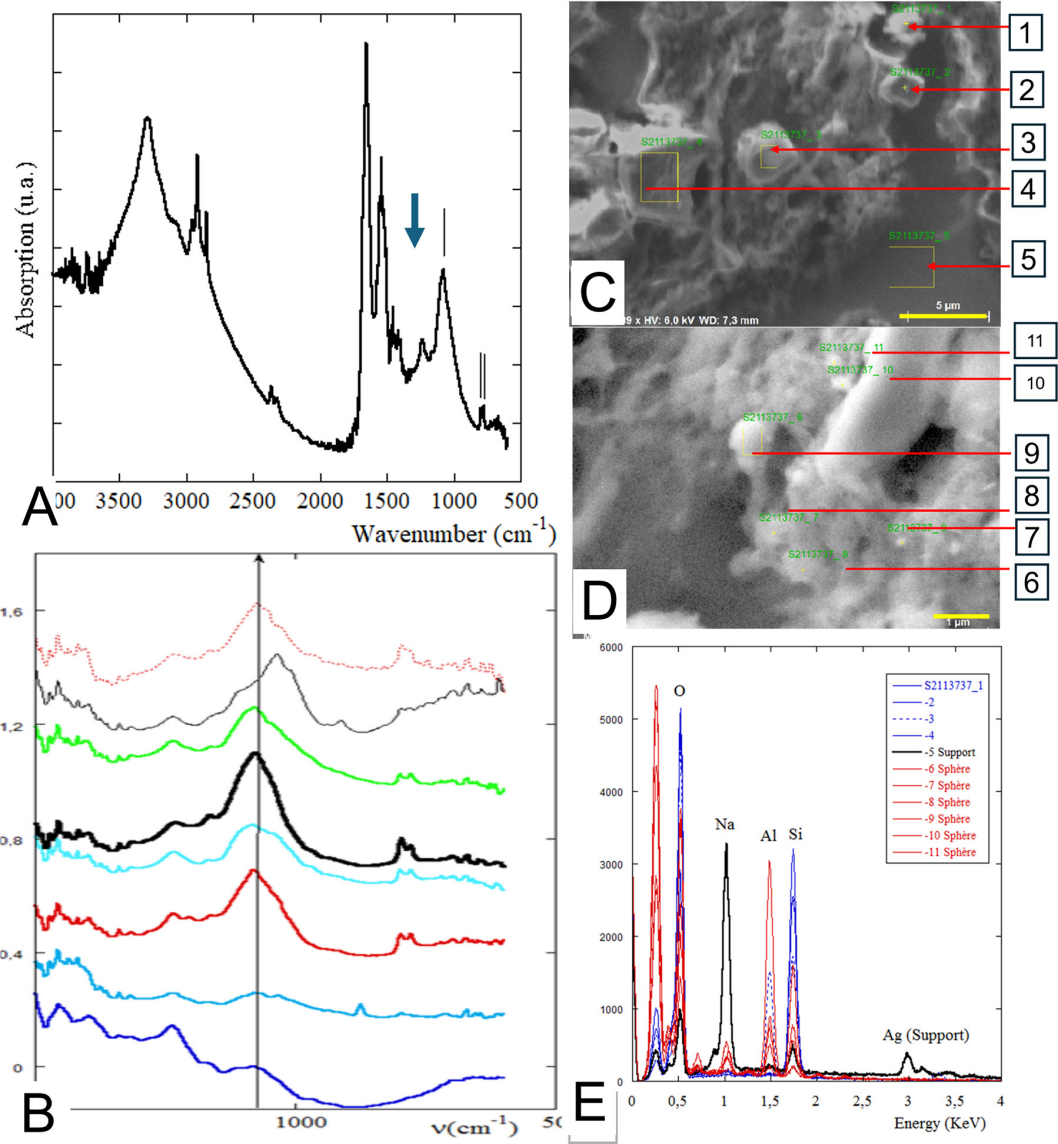
Chemical characterisation of silica particles in mediastinal lymph nodes. (**A** and **B**) The result of an analysis by Fourier-transformed infrared spectroscopy. (**A**) The fundamental Si–O stretching band at ~1100 cm⁻¹ which is the dominant structural band in the infrared absorption spectra of silica (Efimov AM, Pogareva VG (37)) and (**B**) compares the infrared spectra of a selection of deposits from the 17 MLN samples tested. The last two spectra did not show the Si spectrum and were not included in the subsequent SEM study. Correspondence between each spectrum and the sample identification can be seen in online supplemental figure 2 and online supplemental Table 1. (**C** and **D**) The results of an SEM study of an MLN, revealing two distinct morphological types: polyhedral with angular profiles (**C**) and spheroidal (**D**). (**E**) The energy diffraction X-ray analysis of the polyhedral deposits which have spectra restricted to Si and O (numbers 1–4). This is consistent with crystalline silica. The spheroidal deposits have spectra composed of Si and Al (numbers 6–11), which is consistent with aluminosilicates, such as feldspar. (The correspondence between each photograph and the sample identification is reported in online supplemental Table 1). MLN, mediastinal lymph node; SEM, scanning electron microscopy.

FTIR spectroscopy provided an overview of the observed spectra in the analysis process. As shown in figure 3A and a fundamental Si–O stretching band at ~1100 cm⁻¹ associated with silica^37^ was observed in 15 out of 17 (88%) of the analysed samples. No characteristic Si spectrum was detected in two MLN samples (figure 3B and online supplemental figure 2), indicating that the quantitative level of mineral required for detection by this method was not present. It is also important to note that the absorption spectra may be more or less flattened in shape, as illustrated in figure 3B. This indicates the presence of crystalline or amorphous silica particles, as well as aluminosilicates.

**Figure 4:**
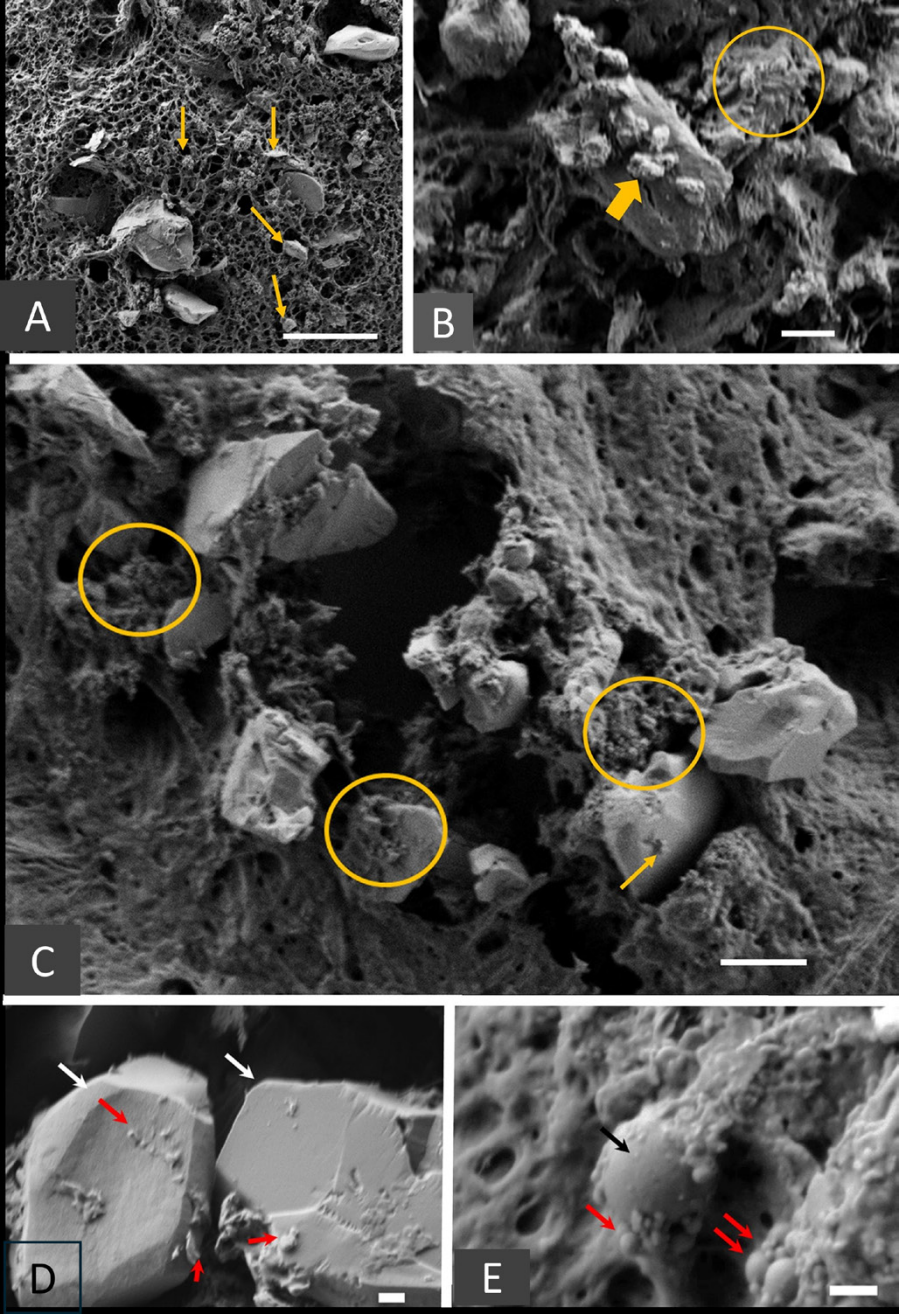
Scanning electron microscopy characterisation of silica particles. (**A–C**) The various physical patterns of particles observed in the mediastinal lymph node samples. Micronic particles are mostly triangular or polyhedral in shape, as are submicronic particles. Arrows indicate submicron and UF particles with angled shapes, which are often associated with larger particles (**B** and **C**). The thick arrow in panel **B** and the circles in panels **B** and **C** indicate clusters of UF particles. Scale bars 2 µm for A, 1 µm for **B**, **C**. (**D** and **E**) show ultrafine nano-sized particles. The particles in **(D**) are mostly polyhedral UF particles (red arrows) similar to the micronic particles (white arrows), while the particles in **(E**) are mostly spherical with a smooth surface (red arrows) similar to the larger particles (black arrows). This suggests a common origin for nanoparticles and larger particles. Scale bar: 200 nm. (The correspondence between each photograph and the sample identification is reported in online supplemental table 1). UF, ultrafine.

SEM observations were conducted on eight MLN samples. The presence of silica-containing mineral particles was confirmed in all these samples, each of which displayed IR bands related to silica or aluminosilicate. Two distinct morphological types were observed: spherical entities and plate-like, cubic or parallelepiped deposits (see figure 3C–E). The EDX analysis of the polyhedral deposits (figure 3C) revealed spectra restricted to Si and O, consistent with crystalline silica. Similar geometric mineral deposits were observed in all eight of the MLN samples analysed, each of which exhibited an angular profile (figure 4). Their size ranged from the micrometre scale to the nanometre scale, with some particles approaching 10 nm in diameter. Figure 4 summarises the different physical patterns of the observed particles. Spherical entities ranging in size from the submicrometre to the nanometric scale were also observed and analysed using the EDX spectrometer (figure 3C). Their observed spectra showed silica and aluminium peaks, indicating a mixture of silica and aluminosilicate particles. The online supplemental table 1 reports the identification of the samples from construction workers shown in figures 3 and 4.

## Discussion

### Summary of key study findings

This simulation was designed to replicate the occupational exposure associated with common tasks in the construction industry, such as cutting or drilling materials that contain silica (eg, concrete kerbs, granite pavement slabs and solid concrete cylinder blocks). This experiment resulted in the release of large quantities of UF particles. Notably, cutting very dense concrete kerbs and granite pavements generated large quantities of UF silica particles, as well as emission peaks of freshly fractured particles. Other reports have indicated that using high-speed tools on various dense materials increases the emission of nano-sized particles, which is consistent with our observations.^38^

Additionally, the analysis of lymph nodes removed during systematic surgical mediastinal clearance (the recommended surgical protocol for treating lung cancer) from construction workers exposed to silica also revealed the presence of nanoparticles corresponding to UF crystalline silica with a polyhedral morphology.

### Limitations and contributions of the study

The experimental protocol used materials and tools that are commonly found on construction sites. However, there are other materials and tools in use in the construction industry, and it is not possible to determine retrospectively whether the patients whose lymph node samples were analysed in this study used the tested materials and tools or, indeed, in what conditions they were used. Nevertheless, construction professionals were consulted to select materials and tools that are typically used under normal site conditions. Similarly, the aerodynamic characteristics of the glove box differ from those of the atmosphere found on a construction site. While these characteristics modify the absolute concentration of RCS over an 8-hour working day, they should not affect the instantaneous emission of particles or their concentration. Therefore, the method of reporting RCS exposure peaks remains valid, as demonstrated during the cutting of concrete and granite paving stones. Furthermore, these characteristics do not affect the presence of UF silica particles, as confirmed by TEM/EDX spectroscopy and X-ray diffraction analysis. These results suggest that particles generated by hand tools during the fracturing process can be inhaled near the respiratory tract of workers. Using the glove box prevented contamination from UF particles in the outside air, such as those from air pollution and co-activities. Therefore, it can be concluded that such aerosols contain highly reactive biological particles that are nanometre-sized and fractured very close to inhalation and that are not even considered by the regulatory methods used to assess exposure to RCS in the workplace. Consistent with our observations, other experiments have shown that the high-speed cutting of various materials results in different nanometric and micrometric emissions.^38^ These results confirm that construction workers can be exposed to UF crystalline silica particles, particularly when using very energetic tools on dense construction materials with high crystalline silica content. These results corroborate the findings of previous publications in similar situations, whether in experimental conditions^18^,^21^ or in the workplace.^4 5 11^

MLN samples were taken from construction workers who had undergone surgical resection for lung cancer, in order to test the hypothesis that the same type of UF silica particles could be identified in conditions of high occupational exposure to silica. This exposure was defined on the basis of ascertaining an individual’s working history using a specialised questionnaire and/or the presence of silicotic nodules in the lymph nodes.^30^ Effective methods were employed to detect Si/O bonds (FTIR) and visualise and analyse UF silica particles in FTIR-positive samples (SEM/EDX), thereby confirming their presence. Although it was not possible to perform X-ray diffraction on this type of sample, the polyhedral morphology of particles containing free silica indicates their crystalline nature. Furthermore, while mineral particles have previously been reported in thoracic lymph nodes, this is the first study to identify UF crystalline silica particles in these nodes in situ. This finding is consistent with the translocation of particles, including UF particles, following deposition in the alveolar spaces.^39^

As expected, due to construction workers’ cumulative and complex exposure to particles throughout their lives, other mineral particles were present in the lymph nodes. It is noteworthy that the recruitment of patients who had undergone lung cancer surgery presented the only opportunity for collecting consecutive thoracic lymph node tissues. Although this procedure may over-represent patients exposed to silica due to its carcinogenic effects, this potential bias does not affect our qualitative conclusion regarding the presence of UF silica particles.

### Consequences for industrial hygiene, epidemiology and clinical practice

Available toxicological data have shown that the biological effects of crystalline silica are amplified by its nanometric size, the fracturing of the crystals themselves and exposure peaks.^22 23^ These findings must be considered when interpreting the results from cohorts of workers in which effects greater than those expected based on dose-response relationships derived from conventional RCS measurements have been observed at certain exposure levels. A comparison of toxicological data for fine and UF crystalline silica is not yet appropriate, because such studies are based on amorphous silica manufactured as nanoparticles.^26^ Nevertheless, a substantial amount of data has recognised the pathogenic role of the physicochemical characteristics of particles of nanometric size.^40^ Furthermore, conventional methods of measuring occupational exposure based on the mass determination of respiratory (fine) particles do not take into account such UF particles.^41^ This could also explain discrepancies between different cohorts of silica-exposed workers with the same level of cumulative RCS exposure.^42 43^

These findings highlight the need for occupational hygienists to supplement conventional RCS measurements with measurements of UF silica particles, and to review protocols for determining OELs. Furthermore, preventive measures must be strengthened during operations likely to emit these particles. Situations in which this occurs in the building industry should also be inventoried in other workplaces where silica exposure can occur.

When diagnostic (mediastinoscopy) or therapeutic (surgical treatment of bronchopulmonary cancers) procedures allow it, using thoracic lymph node samples in clinical practice presents an invaluable means to detect the presence of silica or consequences such as silicotic nodules. These findings should be considered as further evidence of significant past exposure to silica, and they can be used for forensic purposes.

## Conclusion

Abundant emissions of free crystalline nanoscale silica were detected when a Dremel tool was used to cut a concrete kerb and a granite pavement slab. UF silica particles, along with other minerals, have been found in the MLN of construction workers. In light of the suspected adverse effects of nano-sized silica particles, these results highlight the importance of further research in this area. Additional studies are needed, including field work, emission chamber studies and research that characterises human tissue exposed to UF particles. Nevertheless, the data available to date are already sufficient to reinforce the necessity of preventing silica exposure in the workplace, particularly in situations where UF crystalline silica particles can be emitted from freshly fractured materials. The consequences of do-it-yourself activities involving materials that contain silica and tools that are available to the general public should also be considered.

## Supplementary material

10.1136/oemed-2025-110330 online supplemental figure 1

10.1136/oemed-2025-110330 online supplemental figure 2

10.1136/oemed-2025-110330 online supplemental file 1

## Data Availability

No data are available.
