# Supplementary figures and images for: Experimentally recreated workplace environments contain submicron crystalline silica particles, including ultrafine particles, which have been identified in the mediastinal lymph nodes of construction workers
Source: Occup Environ Med. 2026 Apr 21;83(2):e110330. doi: 10.1136/oemed-2025-110330 (PMC13217083; doi:10.1136/oemed-2025-110330)

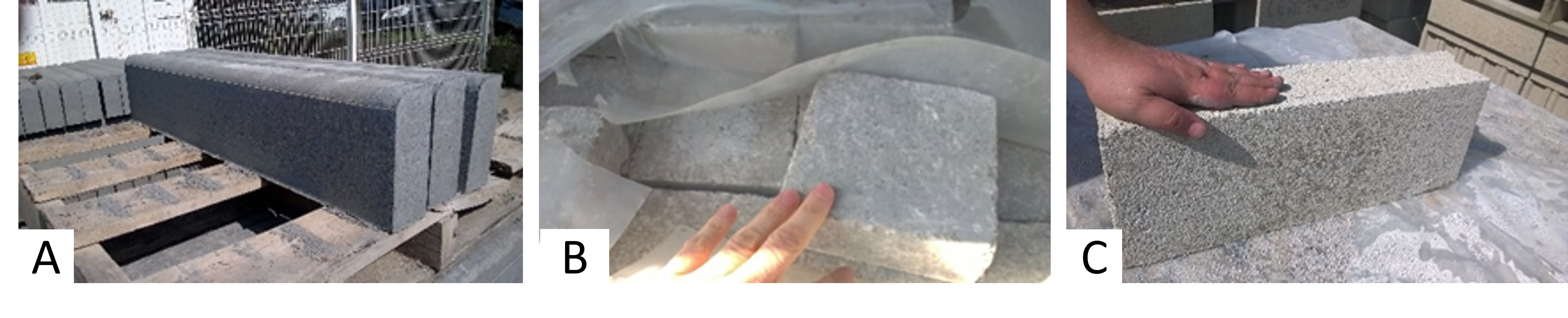

Supplement: online supplemental figure 1 [file oemed-83-2-s001.tif]

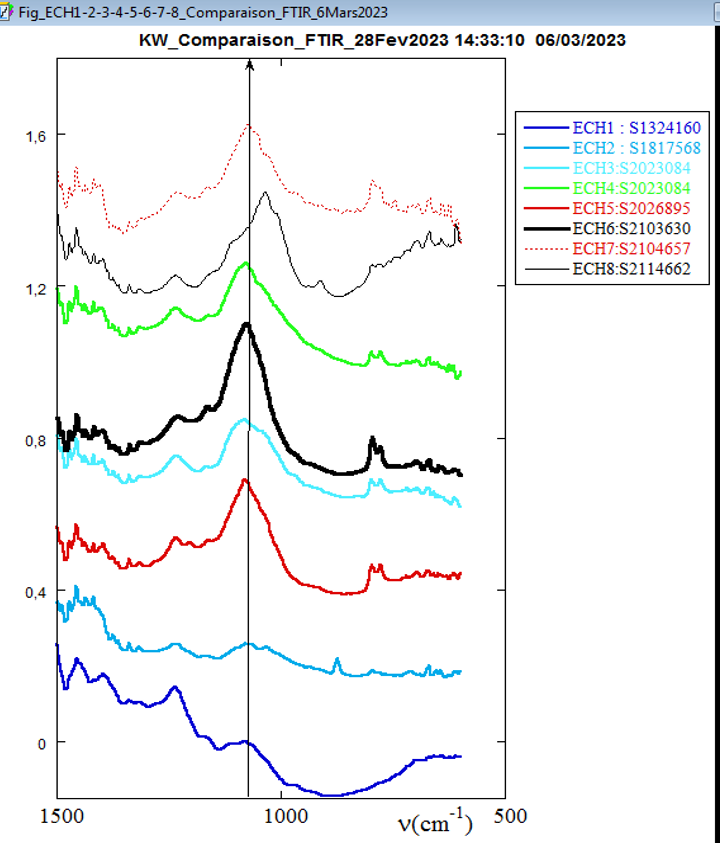

Supplement: online supplemental figure 2 [file oemed-83-2-s002.tif]
