# Supplementary material for: Experimentally recreated workplace environments contain submicron crystalline silica particles, including ultrafine particles, which have been identified in the mediastinal lymph nodes of construction workers
Source: Occup Environ Med. 2026 Apr 21;83(2):e110330. doi: 10.1136/oemed-2025-110330 (PMC13217083; doi:10.1136/oemed-2025-110330)
Supplement: online supplemental file 1 [file oemed-83-2-s003.pdf]

Supplementary material (Figure legends and table)

Experimentally recreated workplace environments contain submicron crystalline silica particles, including ultrafine particles, which have been identified in the mediastinal lymph nodes of construction workers.

1. **Supplementary Figure 1 legend.**

Photographs showing the materials used in the experimentally recreated workplace environments: 1) concrete kerb; 2) granite pavement; 3) solid cinder block. See the "Materials and Methods" section and the "Experimental emissions from stressed construction materials" sub-section.

2. **Supplementary Figure 2 legend**

This figure shows the eight labelled, coloured spectra for the annotated samples in panel 3B of the original Figure 3. The identification of these samples is reported in Supplementary Table 1. The figure clearly shows that six of the spectra exhibit the significant 1100 peak, identifying the presence of silica. In contrast, this peak was not detected in samples 1324160 and 1817568.

3. **Supplementary Table 1. Identification of the samples from construction workers analysed and shown in Figures 3 and 4, regarding to the presence of a high silica exposure and a characteristic Si spectrum by FITR analysis**

A	B	C	D
Figure	Sample number	High silica exposure	FITR
Figure 3A	S2110574	+	+
Figure 3B	S1324160	-	-
	S1817568	+	-
	S2023084	+	+
	S2026895	+	+
	S2103630	+	+
	S2104657	-	+
	S2114662	+	+
Figure 3CDE	S2113737	+	+
Figure 4A	S1722033	+	+
Figure 4B	S1817568	+	+
Figure 4C	S2110574	+	+
Figure 4DE	S2113737	+	+

[A] Figure. [B] Anonymised sample number. [C] High silica exposure according to Brochard et al. (30). [D] The presence of a Si spectrum band is indicated by a plus sign (+).
